# Carbon-based glyco-nanoplatforms: towards the next generation of glycan-based multivalent probes

**DOI:** 10.1039/d2cs00741j

**Published:** 2022-11-23

**Authors:** Javier Ramos-Soriano, Mattia Ghirardello, M. Carmen Galan

**Affiliations:** a School of Chemistry, University of Bristol, Cantock's Close Bristol BS8 1TS UK m.c.galan@bristol.ac.uk; b Glycosystems Laboratory, Instituto de Investigaciones Químicas (IIQ), CSIC and Universidad de Sevilla, Américo Vespucio, 49 41092 Sevilla Spain fj.ramos@iiq.csic.es; c Departamento de Química, Universidad de La Rioja, Calle Madre de Dios 53 26006 Logroño Spain mattia.ghirardello@unirioja.es

## Abstract

Cell surface carbohydrates mediate a wide range of carbohydrate–protein interactions key to healthy and disease mechanisms. Many of such interactions are multivalent in nature and in order to study these processes at a molecular level, many glycan-presenting platforms have been developed over the years. Among those, carbon nanoforms such as graphene and their derivatives, carbon nanotubes, carbon dots and fullerenes, have become very attractive as biocompatible platforms that can mimic the multivalent presentation of biologically relevant glycosides. The most recent examples of carbon-based nanoplatforms and their applications developed over the last few years to study carbohydrate-mediate interactions in the context of cancer, bacterial and viral infections, among others, are highlighted in this review.

## Abstract 2

This review highlights the recent achievements in the field of carbon-based multivalent glyco-nanoplatforms with biological applications.

## Graphical abstract

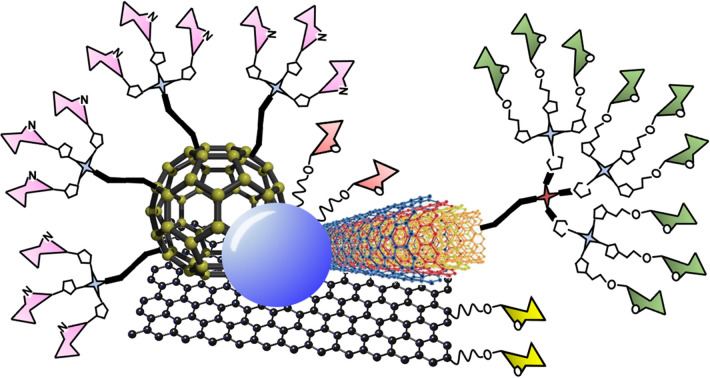

## 1. Introduction

Carbohydrates are ubiquitous molecules found in all cells and organisms and are vital components that mediate many biological, physiological as well as pathological processes,^1^ such as cell growth and differentiation, pathogen infection, tumour progression and metastasis, inflammation, and many others.^2^ Carbohydrate-recognition processes result from interactions with specific proteins such as lectins.^3^ These carbohydrate–lectin interactions are often characterized by high selectivity and low affinity typically in the mM to μM range which in most cases require divalent cations such calcium. This low affinity is compensated in nature by the presentation of multiple copies of the carbohydrate epitopes in a multivalent manner to its receptors to enhance the binding affinity and in many cases the selectivity, in an effect often referred to as multivalency.^4,5^ While this effect is commonly known, the nature of these multivalent interactions is not completely understood from a molecular perspective, due to the complexity and heterogeneity of these binding events. It is proposed that clustering, rebinding, and chelation processes can all contribute simultaneously to the multivalent interaction, however dissecting the contribution of each event individually is very difficult in most instances.^6^ Thus, given the importance of these interactions, to gain a fundamental understanding of protein–carbohydrate mediated processes, the study of multivalent interactions is a topic of much interest in the glycobiology field.

Glycan-functionalized nanomaterials have been developed using a wide range of nanoplatforms that can present glycans in a multivalent arrangement with different architectures, to study multivalent carbohydrate-mediated processes and their interactions with target biological receptors.^7–9^ Moreover, these probes bearing a range of carbohydrate ligands (natural or synthetic ones) have been evaluated in enzymatic studies,^10^ drug delivery applications and in the development of anticancer and antimicrobial therapies.^11,12^ These studies have helped to shed light into the parameters that control carbohydrate–protein interactions and it has been shown that not only the degree of multivalency, but also the type of glycan and conjugation (*e.g.* conjugating linker) and the shape and size of the probe play a pivotal role in how these probes interact with their biological target, which in turns will have an influence in how glycan-conjugates interact in *in vitro* and *in vivo* environments. In recent years, many researchers have focused their efforts towards the relatively less explored carbon nanostructures, such as graphene and their derivatives, carbon nanotubes (CNTs), carbon dots (CDs), and fullerenes as very attractive and biocompatible 2D or 3D scaffolds for the multivalent presentation of carbohydrates.^13^ In this review, we will discuss recent developments in the field of multivalent carbon nanoform-based glycoconjugates and particularly their biological applications.

## 2. Glycographenes

Pristine graphene and its derivatives such as graphene oxide (GO)^14^ and reduced graphene oxide (rGO)^15^ have become attractive platforms due to their high surface area, chemical stability, electrical conductivity and photoelectric properties. Moreover, these carbon based material also exhibit high biological tolerance which has led to their application as functional nanomaterials,^16^ in nanomedicine applications,^17^ microelectronics^18^ and as components of wearable devices,^19^ among others.

Graphene platforms have been functionalised with glycans *via* covalent or non-covalent interactions.^13^ The addition of carbohydrates increases water solubility, colloidal stability and biocompatibility of these carbon-based nanomaterials decreasing the tendency to aggregate in aqueous solutions and imparts the final compound with biological targeting abilities through glycan interactions with their biological receptors.^20,21^ In most cases, glycoconjugation of GO and rGO probes is accomplished *via* non covalent π–π staking interactions between the graphene scaffold and a glycosidic derivative functionalized with an aromatic motif. However, examples of glycan-conjugates where single carbohydrates,^22–25^ or polysaccharides,^26–29^ are covalently bound to either pristine graphene or rGO have also been described. In this section, the most recent examples on the preparation of multivalent glycan-decorated GO and rGO probes and their applications as biosensensors or in the development of antiviral, antimicrobial and anti-cancer therapies are described.

### 2.1. Glycographene probes in biosensing applications

Glycan-functionalised graphene nanomaterials were prepared for the detection of specific multivalent carbohydrate/protein interactions *in vitro* and in complex matrixes.^30^ Glycosides functionalised with aromatic dyes were developed as fluorescent reporters that could be adsorbed on the surface of the graphene *via* π–π interactions. This approach helped to improve the water solubility and concomitantly reduced the cytotoxicity properties of some organic dyes which then allows their use for imaging and sensing studies.^31^ Most examples of glycographene derivatives have found applications in the detection of specific lectins with their known glycan ligand in pair systems such as glucose or mannose/ConA^30^ and galactose/peanut agglutinin (PNA).^32^

These model systems helped to demonstrate *in vitro* the efficiency and specificity of the glycoconjugate-graphene nanoprobes before moving to more complex systems. Mannose-functionalised graphene derivatives can be exploited for the detection of mannose-binding receptors expressed on the cell surface such as *E. coli*, dendritic cells and macrophages; whereas galactose-functionalised graphenes can be used for the detection of galactose-binding asialoglycoprotein receptors expressed on the surface of hepatocytes or peritoneal macrophages.^33–35,36^

**Fig. 1:**
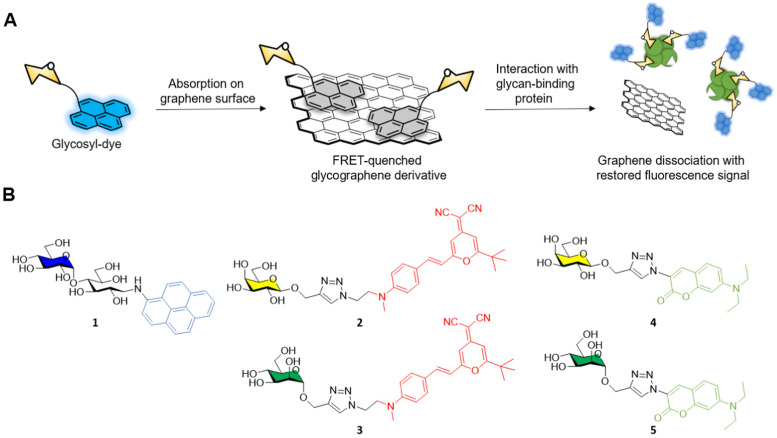
(A) General principle for graphene and graphene derivatives-based FRET fluorescent glycoprobes used for glycan-binding protein sensing. (B) Structure of aminopyrene-1, dicyanomethylene-2 and 3, and aminocoumarin-4 and 5 glycoside derivatives.

The general approach adopted by scientists involves the conjugation of the glycosidic moiety to a fluorescent aromatic reporter able to interact with the surface of graphene derivatives *via* π–π stacking interactions. Upon binding on the graphene surface the aromatic dye fluorescent signal is quenched due to Förster resonance energy transfer (FRET) effects. The subsequent interaction with lectins, or a general glycoside-binding protein causes the dissociation between the glyco-dye and the graphene surface restoring the fluorescence signal which can be detected and quantified to monitor the presence and concentration of specific glycan-binding proteins (Fig. 1A).

In a pioneering work by Chen *et al.*^37^ maltose was functionalised with aminopyrene *via* reductive amination providing the fluorescent glycoderivative 1 (Fig. 1B) which fluorescent emission is quenched *via* FRET effect after adsorption on the graphene surface as described above. In presence of ConA, the lectin binds to the glucose moiety of the maltose-aminopyrene derivative promoting the detachment of 1 from the graphene surface and restoring the fluorescence of the aminopyrene moiety. The system allowed the detection of ConA with a limit of detection (LOD) of 0.8 nM.

The same glycofunctionalisation approach was recently reported by Han *et al.*^38^ for the functionalisation of graphene sheets with the same aminopyrene-maltose conjugate 1. In this case, the team exploited the different atomic lattice given by the self-assembled and densely packed pyrene-maltose layer on the graphene surface for the identification of the binding event with ConA lectin *via* atomic force microscopy (AFM). After graphene surface modification with mannosides, the roughness of the surface adhesion forces can be determined *via* AFM showing a 3-fold enhancement in the adhesion force from 0.66 nN to 1.94 nN for ConA treated graphene. Control experiments with BSA instead of ConA showed no difference in the detected adhesion forces, demonstrating that the protein absorption on the graphene surface was due to a glycan-mediated binding event with a specific lectin receptor.

The He group reported another example of fluorescent glycan-decorated graphene nanomaterias for biosensing applications, whereby galactose and mannose binding proteins could be detected simultaneously.^39^ To that end, dicyanomethylene (2, 3) or aminocoumarin (4, 5) glycoconjugates featuring galactose and mannose motifs, respectively, that gave two distinct emission profiles upon excitation at 430 nm were grafted onto the GO platform causing the FRET-promoted quenching of the fluorescent signal (Fig. 1B). As before, the fluorescence of the dyes was restored upon binding between the protein and the glycan on the GO surface which leads to the dissociation of the glycoconjugate dye (Fig. 1A). In this manner, mannoside and galactoside receptors could be detected with LODs in the naomolar range (ConA, 37 nM and PNA, 44 nM).

**Fig. 2:**
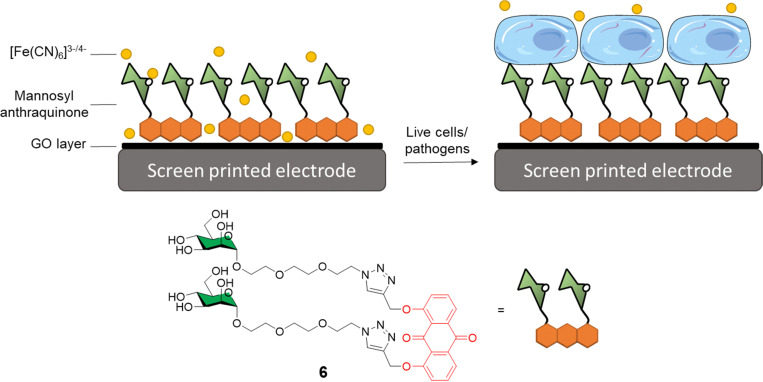
General structure of mannosyl anthraquinone 6 and the glycoconjugate complex grafted on a GO-coated electrode.

Graphene-based electro-sensors modified *via* non-covalent interactions with glycosylated ligands can be used to produce very sensitive detection probes for glycan-binding systems. In this context, Long and co-workers developed anthraquinine glycoconjugates immobilised on GO surfaces for the detection of mannose receptors on the surface of live cells and pathogens.^34,40^ Mannoside anthraquinone glycoconjugate 6 was prepared using copper(i)-catalysed alkyne-azide cycloaddition (CuAAC) ligation, and then grafted onto GO-coated electrodes, which were used to measure the binding affinity between the electrode and either ConA or live cells known to express mannose-specific lectins on their surface *e.g. E. coli* and M2 macrophages cells (Fig. 2). In this experiment, an [Fe(CN)_6_]^3−/4−^ diffusion-to-surface redox process is employed, which detects changes in Fe diffusion caused by the glycan specific binding to its protein receptor with a good linear response over a large range of concentrations. A similar system was also reported by Li *et al.*^41^ In this case, mono and divalent glucose or galactose-modified anthraquinone derivatives graphed on the surface of GO were used for the coating of screen-printed electrodes that could be subsequently employed for the sensing of ConA and PNA lectins, respectively. As expected, the divalent system was able to detect ConA and PNA with a LOD of 16 and 25 nm, respectively, while the monovalent ligand exhibited a higher LOD of 88 and 69 nm, respectively.

These examples of glycan-functionalised graphene nanomaterials demonstrate the potential of such systems as biosensors for the accurate and early detection of cancers and pathogenic infections in the nanomolar range.

### 2.2. Glycographene probes for antiviral applications

The functionalisation of graphene with carbohydrates able to interact with glycan-binding proteins present on viral surfaces can be exploited as a potent tool to monitor, and better understand viral binding recognition and inhibition events, and to hamper the replication of the virus *via* competitive binding with the host cell receptors.

**Fig. 3:**
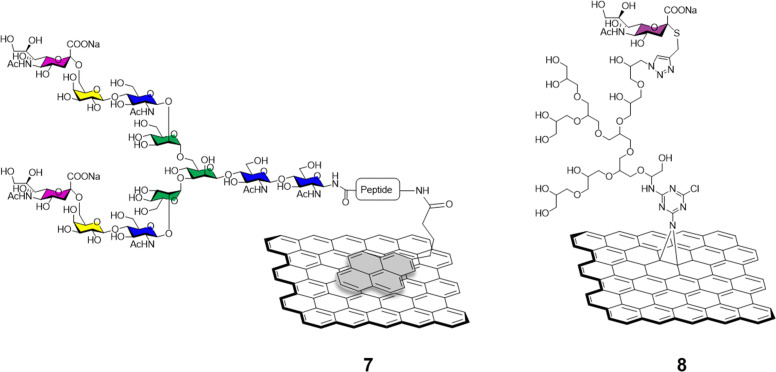
Representative general structures of sialylglycopeptide-coated graphene *via* a pyrene (shown in light grey) 7, and sialylgraphene derivatives 8 which features trGO derivatives modified *via* a triazine moiety bearing a dendrimetic sialic acid conjugate.

The sialic acid viral hemagglutinin receptors binding, and the subsequent sialoside cleavage from the host cell surface by viral neuraminidase represent one of the most studied virus/glycan interactions.^42^ The system is exploited by influenza virus to attach to the sialoglycans present on the host cell allowing virus motility and replication.^43^ Recently, Ono *et al.*^44^ reported the synthesis of glycographene 7 (Fig. 3). Graphene sheets were modified with 1-pyrenebutanoic acid succinimidyl ester and then reacted with sialylglycopeptide providing sialic acid-coated graphene 7. The system was used to build a graphene-based sensor functionalised with sialylglycopeptides in order to study the real time interaction with influenza H1N1 hemagglutinin and neuraminidase enzymes. Moreover, the neuraminidase binding event and its inhibition upon treatment with different commercially approved antiviral drugs such as peramivir, oseltamivir phosphate and zanimivir were also evaluated using AFM and graphene-field effect transistors (FET) measurements, highlighting the potential of the system to detect in real time at a molecular level binding affinities of viral proteins and how antiviral drugs can affect such key interactions.

Bhatia *et al.*^45^ described the use of a covalently-bound sialylgraphene derivative 8 (Fig. 3) based on thermally reduced GO (trGO) platform. The trGO nanomaterial was functionalised *via* a one-pot nitrene [2+1] cycloaddition reaction with 2-azido-4,5-dichloro-1,3,5-triazine followed by polyglycerol modification providing a polyhydroxyl branched intermediate. Controlled mesylation of the hydroxyl functions on the polyglycerol followed by treatment with NaN_3_ furnished azido functionalised nanosheets with differing degrees of azido functionalization. CuAAC reaction with alkyne derivatised sialic acids provided a small library of sialylgraphenes with variable sialic acid surface density *e.g.*, low, medium and highly loaded glyconanomaterials. The glycographenes with medium sialic acid loading revealed to be the most effective agents against influenza A virus by wrapping the virus inside a network of sialyl-functionalised graphene sheets acting as a virus decoy. The competitive binding with sialylated graphene derivatives hampered the virus-cell binding process and consequent viral replication up to a 70.4% against MDCK II cells. These results further demonstrate glycan density is key for receptor recognition events and how important define the optimum glycan presentation parameters are for the design of effective antiviral probes.

### 2.3. Glycographene probes in antibacterial applications

The emergence of antibiotic-resistant microorganisms has led to the development of novel materials and agents as bacterial diagnostic tools and antimicrobials.^46,47^ In this area, glycan-coated graphene and associated materials have also found many applications due to their inert nature and low mammalian cytotoxicity.^48^ The mode of action of many of the graphene-based materials used in antimicrobial applications is due to both oxidative stress caused by the formation of superoxide radical anions (O_2_˙^−^) and bacterial lipid membrane disruption from graphene insertion and phospholipid extraction.^49^

The functionalisation of graphene derivatives with different glycosides can be exploited for an enhanced antibacterial targeting effect, boosting the specificity toward bacterial strains by hijacking the first step on the bacterial infection process which entails the supramolecular interaction between a specific glycan and their protein receptor with the glycographene derivatives.^50^

**Fig. 4:**
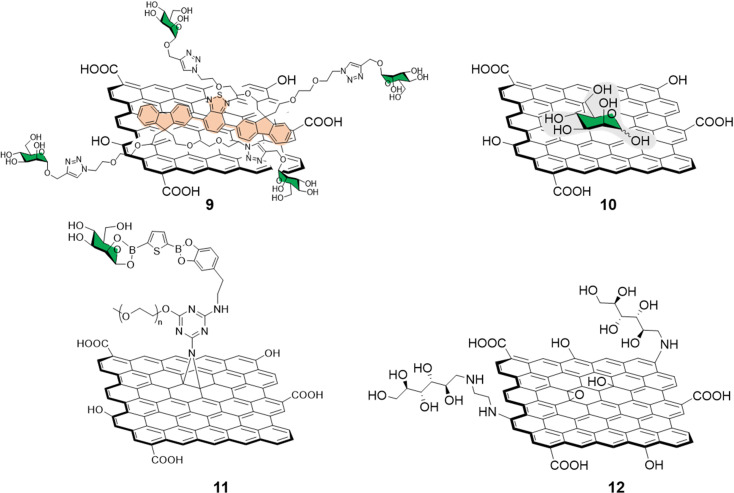
Structure of non-covalently functionalised mannographene derivatives 9 and 10 and covalently bound derivatives 11 and 12.

Different functionalization approaches for bacterial detection have been reported so far, and the most common strategy relies in the non-covalent mannose-functionalisation of graphene derivatives. To that end, Wang *et al.*^51^ reported the synthesis of a tetramannose fluorescent probe 9 which fluorescence signal is quenched upon non-covalent adsorption on GO (Fig. 4). The probe was used for ConA sensing with a LOD of 0.5 nM following the same detection strategy previously detailed in Section 2.1. The team further expanded the applications of this fluorescent probe by testing it against MG1655 and Top 10 *E. Coli* strains. The MG1655 expresses mannose binding FimH lectin (a bacterial adhesion lectin in *E. coli*), while Top 10 does not. FimH expressing *E. coli* were able to bind the mannose probe causing the dissociation from the GO fluorescence quencher to restore the fluorescence. In the case the Top 10 strain, non-specific binding was also observed, albeit in a much more attenuated manner when compared to MG1655. Another development in bacterial sensing came from Bouckaert and Szunerits, the group used a rGO-coated gold interfaces modified *via* passive adsorption of mannose providing glycographene 10 (Fig. 4) and glucosamine (not shown) carbohydrates for the detection and discrimination between different pathogenic *E.coli* strains including UTI89 and LF82, which are responsible for urine tract disorder and Crohn's disease, respectively.^52,53^ A covalent mannose functionalisation of graphene biosensors was reported by Beyranvand *et al.*^54^ through the preparation of GO sheets functionalised with boronic acid moieties capable of binding to *cis*-1,2 or 1,3 diols. Mannose, was then immobilized on the boronic acid-modified GO providing glycoconjugate 11 (Fig. 4) that was used for the selective detection of *E. coli* in complex media such as human blood serum. Using the same probe, different detection techniques were used including UV-vis, fluorescence and differential pulse voltammetry revealing a LODs of 1.20 × 10^3^, 1.15 × 10^3^ and 1.07 × 10^3^ CFU mL^−1^ respectively.

Recently, Chegeni *et al.*^55^ also showed improved antibacterial activity for mannose and glucose decorated GO probes against *K. pneumoniae* and *E. coli* (Gram-negative bacteria), and *B. cereus* and *S. aureus* (Gram-positive bacteria). In their report, functionalisation of GO with ethylenediamine and subsequent decoration with unprotected mannose *via* reductive amination of the carbonyl function gave glycoconjugate 12 (Fig. 4). The team attributed the enhanced antimicrobial activity against all the bacteria species taken in consideration to lipid membrane disruption and oxidative stress.

**Fig. 5:**
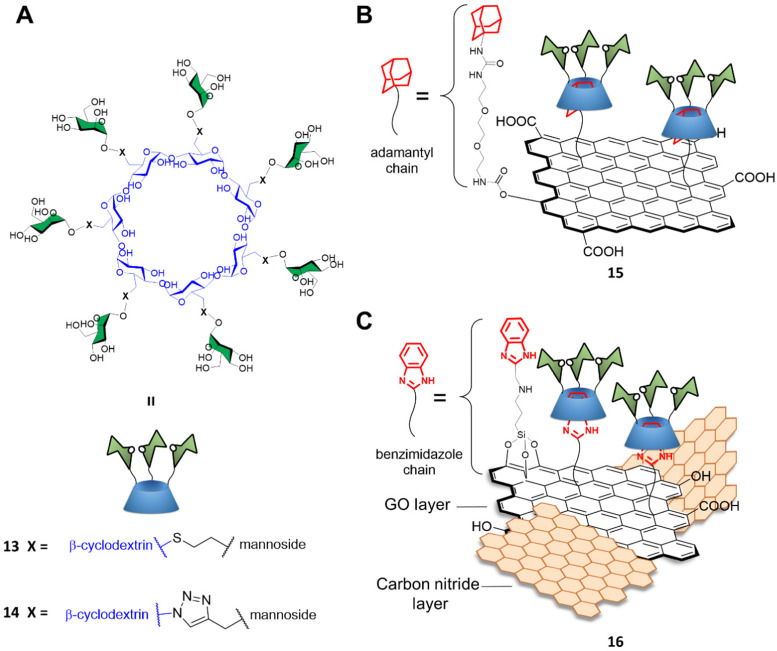
(A) Structures of heptamannosylated β-cyclodextrin 13 and 14; (B) supramolecular assembly of cyclodextrin 13 on adamantyl-functionalized trGO to form 15, and (C) supramolecular assembly of cyclodextrin 14 on benzimidazole-functionalized trGO/carbon nitride composite to form 16.

Graphene-based materials can be activated using IR irradiation to generate heat. This effect has been exploited for photothermal therapy (PTT) applications where light is converted into heat *via* the action of a photothermal sensitizer with great spatial specificity, and where a highly localized temperature increase can inactivate the surrounding cells nearby the probe without damaging any off-target tissue.^56^ Indeed, trGO and rGO platforms can absorb near IR radiation to generate localized temperature increases which can cause damage to surrounding cells where the materials are consigned. To that end, Seeberger and Haag described a supramolecular mannose-functionalized trGO structure and its use against *E. coli*.^57^ β-cyclodextrin 13 was prepared by linking position C6 of the β-cyclodextrin glucosides to 7 mannoside units through a thioether linking moiety (Fig. 5A). A diethylene glycol linker terminated with an adamantyl moiety was employed as an anchor for the supramolecular assembly of heptamannosylated β-cyclodextrin 13 onto trGO to generate 15 (Fig. 5B). The team showed that 13 was able to interact with the *E. coli* surface *via* mannose-FimH receptors to induce bacteria agglutination. Furthermore, the team found that, while non-irradiated bacteria could still form large bacterial colonies after incubation with 15, >99% of bacteria were efficiently killed by IR-promoted PTT upon irradiation at 785 nm for 10 minutes, which led to a temperature increase to *ca.* 70 °C. Moreover, the team demonstrated that the bacteria captured and agglutinated within the trGO-cyclodextrin complex could then be released by the addition of sodium adamantine carboxylate causing the competitive detachment of the cyclodextrin moiety from the trGO scaffold. A similar strategy for bacterial capture and release systems were developed by the Yu and Chen group using a mannose-functionalised β-cyclodextrin reporter for binding to ConA and fimbriated *E. coli.* The team prepared the probe through a layer-by-layer deposition on a gold platform terminating with an adamantly surface decoration which allowed the final functionalization with mannose-functionalised β-cyclodextrin. The group reported the use of competitive adamantly-based^58^ or light promoted^59^ β-cyclodextrin detachment from the platform, creating a *E. coli* catch and release system able to undergo multiple cycles without loss of activity.

These strategies showed great specificity and on-demand bacteriostatic properties demonstrating the potential of these type of materials in the fight against antibiotic-resistant bacteria.

Recently, Wei and co-workers developed a novel mannose-functionalised β-cyclodextrin conjugated on the surface of GO/carbon nitride (CN) nanocomposites as antibacterial agent.^60^ In this case, the β-cyclodextrin 14 was prepared by linking at position C6 of the β-cyclodextrin glucosides, as before, but to seven mannoside units through a triazole linking moiety (Fig. 5A). The GO/CN composite was prepared using standard sonication methods followed by surface functionalization with 3-aminopropyltriethoxysilane and 2-chloromethylbenzimidazole, which act as anchoring points for the subsequent functionalization with mannose-functionalised β-cyclodextrins 14 providing the glycofunctionalyzed nanocomposite 16 (Fig. 5C). The use of GO/CN composites exploits the synergistic effect of the photo thermal properties of GO in combination with the photodynamic properties of CN, which is a nanomaterial able to produce reactive oxygen species (ROS) under visible light irradiation. The novel nanocomplex is able to specifically target mannose-binding bacterial cells, reducing phototherapy off target effects, and killing the bacteria *via* combined PTT and photodynamic therapy (PDT). Dual irradiation at 636 and 808 nm elicited high ROS production and photothermal effect respectively, resulting in high antibacterial activity killing the 99% of the *E. coli* mannose-binding cells (strain CICC 20091). On the other hand, a lower antibacterial effect was reported for non-mannose-binding *E. coli* bacteria (strain CICC 10003) and *S. aureus* achieving an 82% and 71% of bacterial killing, respectively.

Cationic polysaccharides *e.g.* chitosan, can also be used to decorate graphene surfaces *via* ionic interactions to generate novel material composites with unique physicochemical properties.^61,62^ It has been recently shown that the complexation of GO and rGO with chitosan strongly improved mammalian biocompatibility of graphene derivatives, while increasing the antibacterial properties of chitosan.^63^ The positively charged chitosan causes disruption of the negatively charged bacteria cell wall which makes chitosan-based materials promising candidates for biomedical applications.^64^ A similar study was reported by Liu *et al.*^65^ where chitosan-modified GO was used for the electrochemical detection of glucose in solution down to a detection limit of 20 μM. In a recent example, Rostami *et al.*^66^ described the preparation of a sharkskin-mimicked GO/chitosan membranes. This novel material significantly reduced *E. coli* and *S. aureus* biofilm formation while exhibiting high biocompatibility and low to no toxic toxicity towards mammalian cells. More recently, Rahnamaee *et al.*^67^ exploited the combined antibacterial properties of chitosan with the ability of rGO for causing bacterial membrane rupture and oxidative stress, to coat titania nanotubes with rGO/chitosan to create a ternary nanocomposite. In this manner, controlled drug release of vancomycin loaded titania nanotubes promoted bone cell viability with the intrinsic chitosan antibacterial properties. Moreover, rGO also decreased the adhesion of bacteria on the surface, while a synergistic effect for long term antibacterial properties was effected by the chitosan. However, polysaccharide-based composite materials are not multivalent in nature and thus won’t be discussed any further in the context of this review.

### 2.4. Glycographene probes in anticancer agents

PTT in cancer treatment relies on the activation of a pro-drug loaded on a nanoprobe that can be activated upon activation typically upon near-infrared (NIR) irradiation. NIR can be applied with great topological specificity to trigger the drug release and induce photothermal killing.^68^ The efficient implementation of graphene derivatives in cancer therapies requires high *in vivo* chemical stability of the drug-loaded nanomaterials and negligible toxicity toward healthy cells. The nanomaterials will also benefit from the functionalisation with specific ligands able to direct the drug toward the cancer cells to avoid off-target effects. Moreover, the nanocarriers need to escape the clearance from the body for a period long enough to deliver the drug cargo to the cancer cells.^69^

Owing to the low cell cytotoxicity of glycan decorated GO-type materials, these types of glyco-composites have found applications in the targeted delivery of anticancer drugs and in the photothermal ablation of tumours. For instance, lactose^70^ and galactose^39,71^ functionalized GO nanostructures were used for the selective delivery of glycan-decorated graphene probes to hepatoma cells, which overexpress asialoglycoprotein lectin receptors that recognise non-sialylated glycans and mannoside-GO and -rGO conjugates afforded probes targeting mannose receptors overexpressed in macrophages and dendritic cells for drug delivery applications.^72,73^ More recently, efforts have been devoted towards improving the preparation of rGO platforms using fucoidan^74^ or l-ascorbic acid,^68^ to replace toxic hydrazine hydrates which are often used as the GO reductive agent. Over the years, the functionalisation of GO and rGO with polysaccharides such as chitosan and dextran or hyaluronic acid (HA) revealed to be an effective strategy for the preparation of composite materials for anticancer applications.^75–84^ However, these systems are not multivalent in nature and thus won’t be discussed any further in the context of this review.

## 3. Glycocarbon nanotubes (glycoCNTs)

Among other carbon nanostructures, glycan-decorated carbon nanotubes (glycoCNTs) have also been developed for biological and medical applications.^13,85–90^ Whilst CNT-based glyconanomaterials^87,91–96^ are not as widespread as other carbon-based nanoplatforms such as [60]fullerenes (see Section 5 in this review), owing to their unique physico-chemical, mechanical and electrical properties, single and multi-walled carbon nanotubes (SWCNTs and MWCNTs, respectively) have become attractive and biocompatible materials as multivalent platforms for the development of glycan-based nanoprobes. Different approaches have been devised for their synthesis and biomolecule functionalization^97–100^ that can be targeted for different biological applications.^101^ Here, the synthesis and biological applications of the most notable examples of CNT-glycoconjugates recently reported will be discussed in this section.

### 3.1. GlycoCNTs as probes to explore lectin binding

**Fig. 6:**
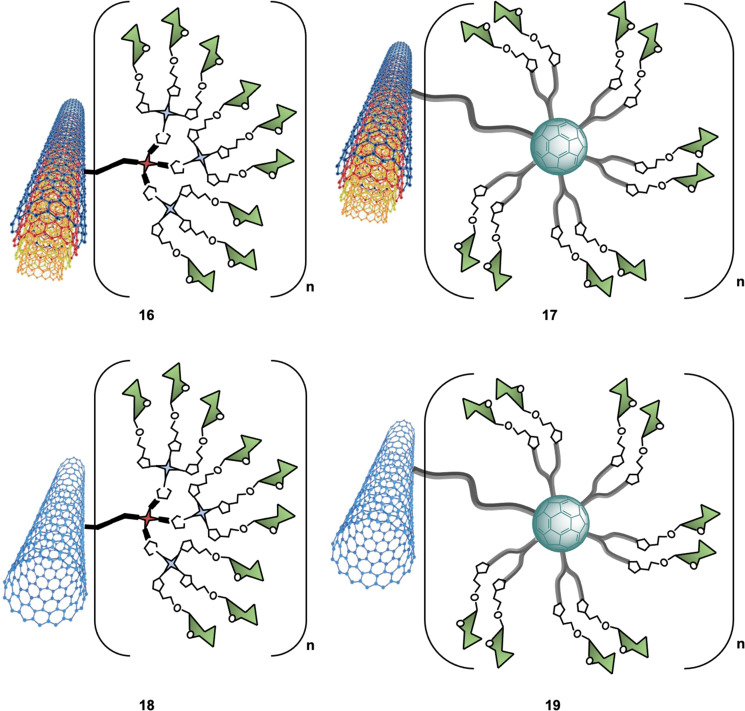
Structures of glyconanomaterials 16–19. For the sake of clarity, a nanotube fragment with one copy of glycodendron or glycofullerene is shown.

C-type lectins, such as DC-SIGN, that recognize carbohydrate structures present on viral glycoproteins associated to several enveloped viruses, and function to enhance viral entry and facilitate infection of cells have also been targeted using nanocarbon-based glycoconjugates. For instance, Martin and co-workers reported the use of multivalent glycoconjugates 16–19 as potent antiviral agents (Fig. 6).^102^ CNTs were chosen as a platform for glycan presentation since the elongated shape of CNTs resembles the filamentous structure of Ebola virus (EBOV) and thus mimic the glycan presentation in this virus. To that end, glycoconjugates 16–19 were thus prepared by a one-pot deprotection/CuAAC click reaction of alkyne functionalised CNTs with azide-functionalised glycodendrons or glycofullerenes (see Section 5 for details on this class of probes).

An important aspect of any glycan carbon-based probe consists in the ability of researchers to structurally characterise the probes in order to correlate the biological function/effect with a well-defined structure. In the context of these hybrid materials, this can be challenging and techniques such as dynamic light scattering (DLS), thermogravimetric analysis (TGA), Raman spectroscopy, Fourier transform infrared spectroscopy (FTIR), X-ray photoelectron spectroscopy (XPS) and transmission electron microscopy (TEM) were required to provide in depth detail of the physicochemical properties of the materials. Furthermore, using a pseudovirus featuring surface Ebola virus glycoprotein (EBOVGP), nanoglycocomposites decorated with mannoside moieties 16–19 were used to evaluate the DC-SIGN-mediated antiviral binding affinity. The team found the 3D architecture of MWCNTs 17 led to a potent EBOV inhibitor, with no significant cytotoxic towards host cells. Moreover, this study revealed that in addition to the size and morphology of the nanoplatform used, the number of multivalent ligands was also important for recognition. Overall, CNFs with tunable size and high biostability properties can be considered very attractive multivalent platforms for the development of antivirals and other applications in biosensing, drug delivery, and pathogen inhibition.

For instance, mannose-covalently modified CNTs were used as a mannose receptor(MR)-targeted delivery system to enhance immune response both *in vitro* and *in vivo*.^103^ This glyconanomaterial significantly improved the ovalbumin (OVA) antigen delivery to macrophages and promoted immune activity in *in vitro* assays, whereas in *in vivo* experiments a dramatically enhanced humoral and cellular immune response against OVA was elicited.

### 3.2. GlycoCNTs as probes in anti-bacterial applications

CNTs-based glycoconjugate scaffolds constructed *via* non-covalent^104,105^ or covalent^106^ bonds have been reported as potent antimicrobial agents. For example, Cid Martín *et al.*^104^ prepared glyconanorings shaped in an abacus-like geometry. The non-covalent supramolecular structures were self-assembled as a result of mannose polymerization of 20a on SWCNTs upon UV irradiation. These water stable 1D-mannose-coated SWCNT 20b were characterized using conventional techniques such as DLS, AFM, TEM, NIR and Raman spectroscopy. Moreover, using an enzyme-linked lectin assay (ELLA), the team observed a significant cluster glycoside enhancement in binding towards ConA for glycoCNT 20b with an over 2340-fold increase in relative binding potency to mannose binding lectin ConA when compared to the monovalent methyl α-d-mannopyranoside.

**Fig. 7:**
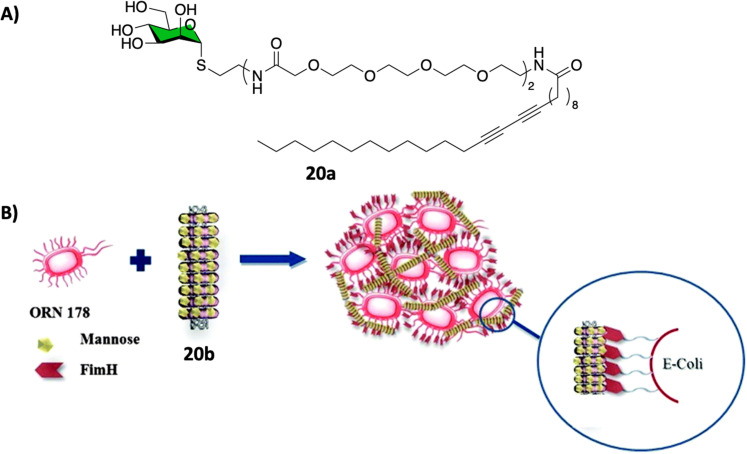
(A) Chemical structure of the polymer 20a that forms the nanorings. (B) Schematic representation of the FimH adhesin promoted specific interaction of *E. coli* with 20b. Reproduced with permission from Royal Society of Chemistry.^104^

These results were further confirmed *in vitro* using an agglutination assay of the enterobacteria *E. coli* type 1 fimbriae (Fig. 7).^104^ In this experiment, mannoCNT 20b was evaluated against ORN178 and ORN208, two *E. coli* strains which differ in whether the bacteria expresses or not the FimH mannose receptor on the surface. The team found that 20b was able to bind selectively to FimH-presenting *E. coli* type 1 fimbriae agglutination as demonstrated by high resolution TEM images and proliferation. No bacterial growth inhibition was observed for FimH-deleted strain, which demonstrate that the interaction is lectin specific event. Subsequently, a similar study by Khiar *et al.*^106^ using mannose or lactose covalently functionalised SWCNTS demonstrated that only mannosyl conjugates were able to agglutinate and inhibit the bacterial growth of *E. coli*, while the corresponding lactose-coated 1D-carbon nanotubes showed no formation of bacterial aggregates as expected since lactose is not a ligand for FimH.

Collectively, these results show how glycan-coated SWCNTs have great potential as a new class of antiadhesive agents and as antimicrobials.

### 3.3. GlycoCNTs as probes in anticancer applications

Glycan-decorated CNTs have also been used in anticancer applications. Taking advantage of the overexpression of the hyaluronic acid (HA)-binding receptors such as cluster determinant 44 (CD44) on the surface of malignant cells, which are known to bind HA glycans, HA-functionalized smart MWCNTs were developed as anticancer drug nanocarriers to encapsulate doxorubicin (DOX) for targeted delivery to cancer cells overexpressing CD44 receptors^107–110^ or as tumor-targeting MRI contrast agent when combined with Gd^3+^.^106^

**Fig. 8:**
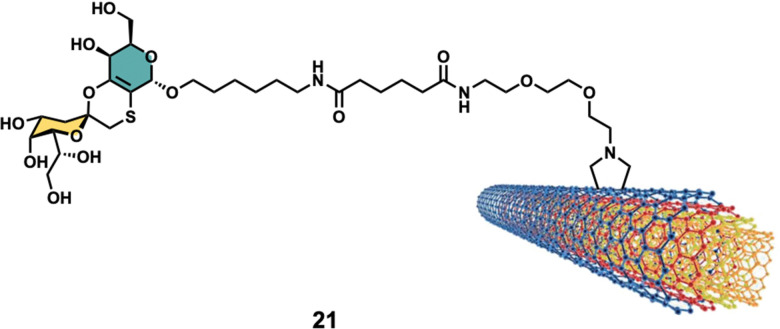
Structure of glyconanomaterial 21. For the sake of clarity, a nanotube fragment with one copy of glycoconjugate is shown.

Other interesting example was reported by Richichi *et al.*^111^ where GM3-lactone mimetics were used to decorate MWCNTs as inhibitors of melanoma-associated metastasis (Fig. 8). To that end, nanosized glycoconjugate 21 was evaluated as an inhibitor of A375 human melanoma cell metastatic processes by monitoring cellular adhesion, migration and invasiveness properties. The *in vitro* results for compound 33 showed a disruptive effect with A375 cell adhesion properties with no general cytotoxicity. Moreover, strong cellular migration and invasiveness inhibition of melanoma cells were also observed. These results further highlight the promising potential for future applications of glyco-MWCNT against melanoma.

## 4. Glycocarbon dots (glycoCDs)

Carbon dots (CDs) represent a novel class of carbon-based fluorescent nanomaterial which have been hailed as an exciting alternative to fluorescent metal-containing nanoparticles thanks to their ease and low cost of preparation, low toxicity and outstanding photoelectric and physico-chemical properties.^112^ CDs are quasispherical nanomaterials with a typical size of 10 nm or below that possess great chemical stability and high-water solubility. Their fluorescent emission can be easily tuned as a function of the reaction conditions and type of precursors used in the synthetic procedure.^113^ Since their serendipitous discovery in 2004 by Xu *et al.*^114^ CDs have found a growing number of applications across many scientific disciplines including semiconductors,^115,116^ biomedicine,^117,118^ catalysis,^119^ sensing and functional materials,^120,121^ and even in the agricultural field.^122,123^

Among the many carbon sources used for bottom-up synthetic approaches for the synthesis of CDs, carbohydrates and their derivatives have been used as a green and cheap source of CDs precursor through different synthetic procedures,^124^ nonetheless, the harsh synthetic procedures used during CDs synthesis are likely to degrade the structure of the carbohydrate precursors. Therefore, the post synthetic functionalisation of CDs with glycosides represents the best option for the preparation of CDs with a well-defined chemical structure on the surface of the nanoparticles. As mentioned above for other nanoforms, the carbohydrate functionalization of CDs confers to the nanoparticles an enhanced bioavailability and therefore a reduced toxicity. This feature was exploited to deliver non-toxic CD-glycoconjugates for cellular labelling and sensing applications. However, to the time writing the delivery of glycoCDs toward biological targets that express carbohydrate receptors in combination with PTT or PDT effect proper of certain CD cores has not been reported and represents an exciting field of research for novel applications of CDs derivatives. Herein, we will describe the most recent advances in synthesis and applications of carbohydrate-functionalized CDs and their applications as antibacterial and anticancer agents.

### 4.1. GlycoCDs as probes in cancer sensing applications

CD nanoparticles have found a great number of applications as anticancer agents. Indeed, their tunable fluorescence emission profiles,^125^ their versatility as anticancer agents through PTT,^126^ PDT,^127^ as nanocarriers for drugs^128^ and gene delivery,^129^ and as immunostimulants^130^ have revealed these materials to be an ideal tool for cancer theranostics. Moreover, the use of key glycan epitopes decorating the surface of CDs can improve the cellular targeting selectivity toward malignant cells expressing lectins and in general glycan-binding receptors (*e.g.* hepatocyte and macrophage cells expressing galactose or mannose receptors, respectively).^131^ While to date, there are very few applications of glycan-coated CDs as cancer cell killing agents,^193^ their use as fluorescent probes for cancer cell imaging has been more extensive.

Among the choice of biologically-relevant glycan epitopes, previous studies by the Galan group demonstrated that lactose represents one of the best moieties to improve cellular internalisation and compartment of quantum dot (QD) nanoparticles.^132^ The screening of a library of mono to trisaccharide-coated nanoparticles revealed that a glycan surface functionalisation improved the bioavailability of QDs and that coating with lactose provided higher uptake in cancer cells. This feature was implemented by Hill *et al.*^133^ in CD model systems, where the CDs’ cellular internalisation was improved by the functionalization with lactose moieties. The core nanoparticle scaffold was built starting from glucosamine hydrochloride (GlcNH_2_·HCl) and 4,7,10-trioxa-1,13-tridecanediamine (TTDDA) precursors *via* three-minute microwave-promoted solvothermal synthesis providing the CD core nanoparticles with high concentration of amine functions on the CD surface. The blue fluorescent amine-coated CD core showed excitation-dependent emission and an average diameter of 2.45 nm with a sp^3^-enriched crystalline core.

**Fig. 9:**
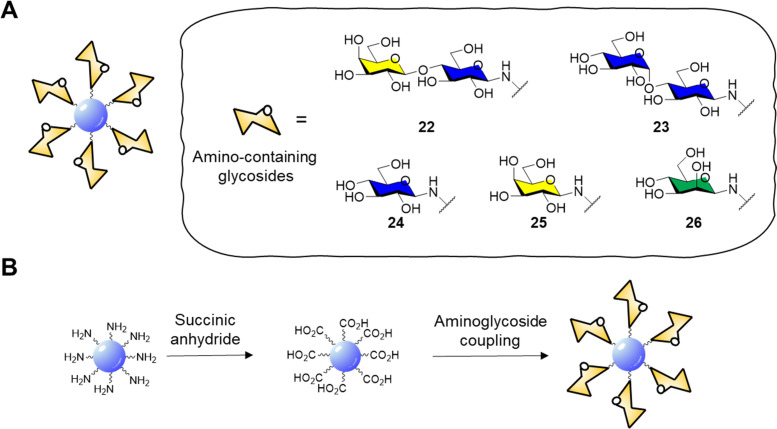
(A) General structure of glycosidic carbon dots and structure of amino-containing derivatives 22–26; (B) scheme for the functionalisation of CD core to aminoglycoside derivatives.

Surface functionalisation of CD cores with glycosidic moieties was then easily achieved *via* a two-step process: first the CD surface was modified with succinic anhydride to provide CDs with acidic moieties on the surface, then carbonyldiimidazole (CDI) activation and coupling with aminolactoside provided lactose-functionalised CDs 22 (Fig. 9A and B). NMR spectroscopy could be used to confirm the successful surface functionalization procedure by showing the presence of characteristic resonance peaks for the carbohydrate as passivating agents. Confocal fluorescent microscopy showed good internalisation of 22 in HeLa and MDA cells and displayed a diffuse localisation within the cell. In a follow-up study, the same group reported the synthesis of blue fluorescent acid coated CDs in a one-pot reaction *via* microwave-promoted solvothermal synthesis using glucosamine hydrochloride and an amino acid precursor.^134^ The core acid-coated nanoparticles revealed high biocompatibility against MDA cells. Confocal microscopy studies revealed a passive internalisation of the nanoparticles through the cell membrane. Moreover, the CDs were also functionalised with lactose units as described for glycoCD 22 and following NMR quantification it was found that these materials had a higher glycosidic density compared to 22.

GlycoCDs biocompatibility and interactions with biological targets and receptors is strongly dependent on how the carbohydrate moieties are presented on the surface shell of nanoparticles and CDs are no different. Full structure characterization is key to understand how the probes interact with their receptors, however working on the nanoscale with platforms with a heterogeneous surface composition can be very challenging. In addition, the type of carbohydrate may influence the electronic surface state of nanoparticle and therefore their photoelectric properties. In this sense, Swift *et al.*^135^ reported the synthesis of a set of carbohydrate-coated CDs 22–26 (Fig. 9A) and demonstrated that the type of glycan moiety influences the three dimensional structure and fluorescence of the CD probes. This study helped explore how glycans with differing stereochemistry (axial and equatorial hydroxyl groups) and size (mono- and disaccharides) are distributed on the CDs surface and how they can influence the CD fluorescence. Furthermore, AFM images revealed a varying morphology with a uniform glycoside shell for glucose, mannose and lactose derivatives whereas galactose and maltose coating resulted in inhomogeneous surface coverage. More recently, Mulkerns *et al.*^136^ developed a practical strategy, based on measuring the refractive index of nanoparticles, to determine the ratio of the volume of the functionalisation layer with regards to the particle volume, *e.g.* CDs in suspension based on refractive index mesurements. This non-destructive optical method can detect differences in surface functionalisation or composition of nanometre-sized particles, making the approach ideal for *in situ* industrial particle characterisation and biological applications.

The cytotoxicity of naked CD core material, as well as the CD intermediates functionalised with succinic anhydride and lactose coated CD 22 was also evaluated against HeLa (human cervical) and MDA-MB-231 (human breast) cancer cells at different concentrations for 1 h, 1, 3 and 7 days using AB/Calcein, which was used to assess cell reductive metabolism. In MDA cells, both naked CD core nanoparticles and the succinic anhydride derivatives showed altered metabolism at concentrations above 250 μg mL^−1^ with no significant cell death. On the other side, 22 showed improved biocompatibility and no differences were observed between the treated and untreated control cells. In HeLA cells, naked CD core nanoparticles showed enhanced cell death at concentrations over 250 μg mL^−1^ while succinic anhydride derivatives and 22 showed altered metabolism above 10 μg mL^−1^. Diffuse cell internalization of glycoCD 22 was observed after 2 hours of exposure validating the use of carbohydrate-coated CDs reduce the overall cytotoxicity of CDs while maintaining the fluorescent properties needed for imaging applications.

**Scheme 1:**
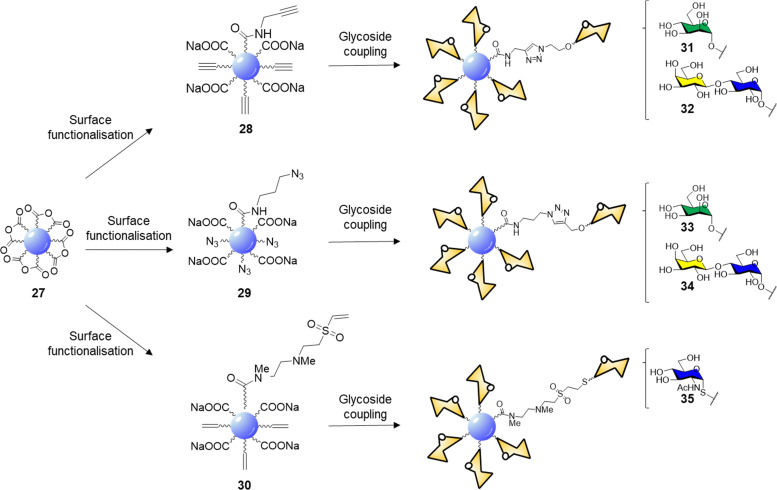
Surface functionalisation of 27 to alkyne 28, azide 29, and vinylsulfone 30 derivatives, and glycan coupling to furnish glycoCDs 31–35.

CDs prepared from citric acid represent one of the most common class of CDs prepared *via* bottom-up synthesis.^137–139^ Santoyo-Gonzalez's group reported the synthesis of blue emitting acidic anhydride CDs 27*via* thermal treatment of citric acidic.^140^ The CD synthesis proceeded through simple thermal decomposition of citric acid. The authors hypothesized that the active anhydride species formed on the CD surface may undergo direct amidation to generate functionalised CDs without the need for coupling agents (Scheme 1).

Indeed, reaction with propargylamine, 3-azido-1-propylamine and *N*,*N*′-Dimethylethylenediamine (DMEDA)/divinyl sulfone furnished the respective intermediates 28–30. XPS and elemental analysis confirmed the effective surface passivation and a four times higher degree of functionalization for the 29 over 28 was observed. In the case of vinyl sulfone derivative 30, the reaction proceeds *via* anhydride amidation and consecutive Michael addition. Final glycan functionalization proceeded *via* microwave assisted CuAAC in case of 28 and 29 with the respective azido or alkyne glycoside derivatives or *via* Michael addition of the thioglycoside derivative on the electron-deficient vinyl sulfone moieties (Scheme 1). Carbohydrate loading was confirmed by FTIR analysis and phenol-sulphuric acid assay.

The lectin binding ability of glycoCDs 31–35 was evaluated *via* ELLA assay using ConA, PNA and WGA for mannose, lactose and GlcNAc coated CDs respectively. The multivalent glycan-CD presentation showed from 2 to 3 orders of magnitude increased in binding affinity than the corresponding free carbohydrates. The cytotoxicity of glycoCDs 31–35 was evaluated against HeLa, CHO-k1 (Chinese hamster ovary), RAW264.7 (murine macrophage) and CT26.WT (murine colon cancer) cell lines showing low toxicity at concentrations up to 250 μg mL^−1^. Furthermore, glyconanoparticles 31–34 showed enhanced uptake when incubated with the cell lines expressing the specific glycan receptor (mannose receptors in RAW264.7 and lactose receptors in CT26.WT cells) validating the use of CDs as multivalent platforms for glycan mediated applications.

An alternative functionalisation approach of citric acid based-CDs was presented by Cooper *et al.*^141^ in 2020. The CDs surface was initially passivated using of 3-glycidyloxypropyltrimethylsilane providing a reactive epoxy moiety that undergoes ring opening reaction upon reaction with a nucleophilic aminoglycoside derivative such as for compound 22 to generate lactose functionalised CDs. Lectin microarray studies showed that lactose-CD conjugates were able to bind lactose and galactose binding lectins on the array surface. Moreover, in agreement with the previous studies, the lactose functionalisation of CDs improved the cell viability toward HeLa cells and confocal analysis demonstrated a lysosomal localisation for lactose-CDs confirming that the glycan coatings can improve the bioavailability of glycoCDs for imaging applications.

Despite the many applications these new type of materials have found so far, the internalisation mechanism of glycan coated CDs are still unclear and further studies are required to assess if internalization is a receptor mediated or passive uptake mechanism. Collectively, the glycan coating of CDs is a valuable method to improve cellular uptake, bioavailability and cytotoxicity profile of CDs. Moreover, the use of glycan-coated CDs for PTT, PDT or selective drug delivery to cancer cells still remain relatively underdeveloped and offers new and exciting opportunities to generate valuable nanotools for cancer therapies.

### 4.2. GlycoCDs as probes in antibacterial applications

CDs have emerged as a promising class of nanomaterials for the detection and inactivation of different bacterial species, the general bacteriostatic or bactericidal activity of CDs is attributed to physical bacterial membrane damage, cell wall destruction, inactivation *via* PTT and direct or light promoted generation of ROS, and DNA and protein damage through the PDT.^142,143^

The specific interaction between the carbohydrate receptor expressed on the surface of bacterial cells and glycan-coated CDs represent a strategic opportunity to enhance the interaction and specificity of CDs with bacteria. Lai *et al.*^144^ reported an improved synthetic approach for the preparation of mannose-CDs to interact with the FimH mannose binding lectin present on *E. coli* cell surface. The new synthetic method was developed in a stepwise manner starting with CD formation *via* thermal decomposition of ammonium citrate, followed by dry coupling with mannose. The coupling reaction worked without the need of a coupling reagent by heating the CDs and free mannose at 180 °C in a crucible. The reaction most likely occurred *via* dehydration coupling between the carboxylic acids on the CD surface and hydroxyl groups of the coating agent, in this case the carbohydrate. The CDs were able to fluorescently label *E. coli* with excitation dependent emission showing a limit of detection for labelled bacteria in PBS buffer solution of 100 CFU mL^−1^.

**Scheme 2:**
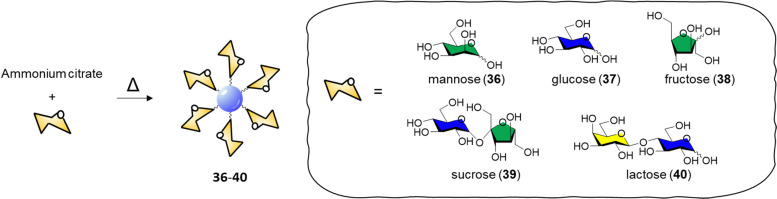
Synthesis of glyco CDs 36–40*via* thermal decomposition of ammonium citrate and the respective carbohydrate.

In 2015, Huang and co-workers reported the synthesis of glycoCDs 36–40 using of a small library of carbohydrates *via* a dry heating process (Scheme 2).^145^

The adopted approach consists in the thermal decomposition of a solid mixture of ammonium citrate and the chosen carbohydrate in a one pot reaction. The ability of these glycan-coated CDs to label bacteria cells was screened against *E. coli*, *P. vulgaris*, *S*. *aureus* and *B*. *subtilis* strains. Only the *E. coli* strain were labelled by the mannose-CDs 36, due to the presence of FimH lectins expressed on the surface of the bacterial pili. No significant labelling was achieved with other glycan-decorated CDs. Titration experiments demonstrated that only free mannose or glucose are able to compete with the binding of mannose-CDs 36 to *E. coli* showing a 90% and 8% fluorescence decrease in the presence of a 250 mM solution of mannose and glucose, respectively. Labelling of *E. coli* in the presence of different bacterial strains showed a 1000-fold affinity for *E. coli* over the aforementioned competing bacteria with a limit of detection for *E. coli* labelled with mannose-CDs 36 of 450 CFU mL^−1^. The selectivity and sensitivity of Man-CDs for *E. coli* was exploited for the successful detection of the bacteria in different matrices (tap water, apple juice, human urine) revealing a minimum detectable concentration in all the three matrices of approximately 10^3^ CFU mL^−1^.

## 5. Glycofullerenes

Many bioactive carbon nanoforms have been developed over the last few years, among those, glycofullerenes, and in particular hexa-substituted glycofullerenes have attracted much attention as a biocompatible carbon nanoplatform for the multivalent presentation of carbohydrates owing to its octahedral symmetry and globular structure, which allow the homogenous 3D distribution of the carbohydrates around the C_60_ fullerene core.^146,147^ Moreover, fullerene C_60_ post-functionalizable hexakis-adducts allow the simultaneous grafting of twelve groups which offers a significant advantage over other carbon-based platforms for the fast construction of higher degree of multivalency by using dendrimers which leads to better define glycan presentation.^148^ For synthesis and other examples of monoadduct of fullerene C_60_ glyco- and peptide-conjugates, the interested reader is directed to a recent review reported by Zhang and co-workers.^149^

### 5.1. Glycofullerenes as probes in antiviral applications

Glycoconjugate-based strategies developed against viral infections aim to mimic the viral surface carbohydrate cloak which is often of globular geometry. These glycan-probes are designed to can block the infection process by disrupting the interaction between the cell-surface receptor and the corresponding native glycan.^150^ In this context, glycofullerenes with T_h_ symmetry have been extensively exploited over the last decade for the preparation of multivalent glycoconjugates.^151^

**Fig. 10:**
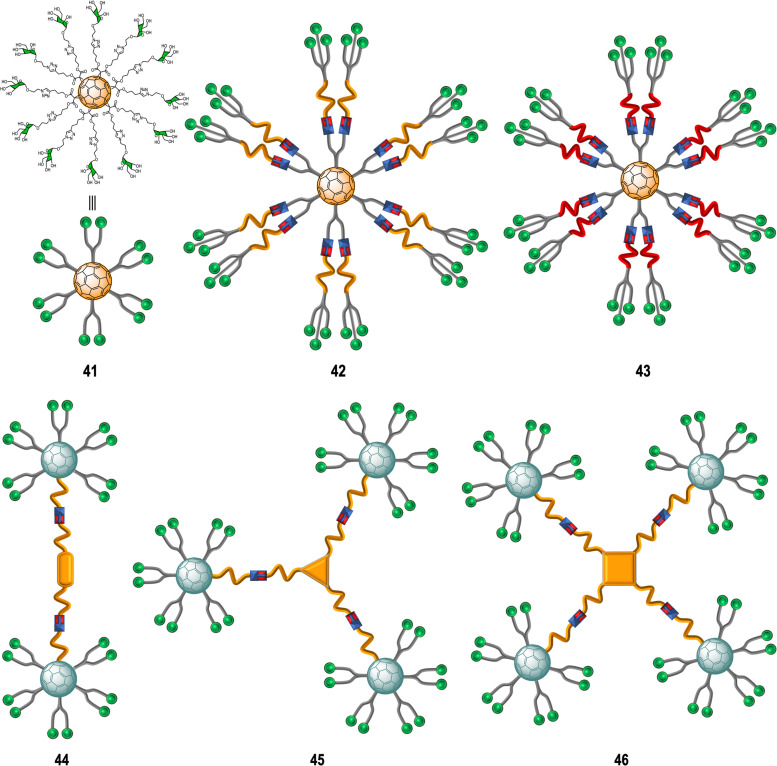
General representation of glycofullerenes 41–46.

Rojo, Martin and co-workers developed a straightforward strategy based on the CuAAC “click” reaction whereby twelve glycan-motifs are simultaneously conjugated to alkyne-substituted hexakis-adducts in a regioselective and efficient manner, in just a few steps, with good yields.^152–154^ Using this strategy, the team's efforts developed “sugar-balls” as inhibitors of emergent viruses such as Zika (ZIKV), Ebola (EBOV) and Dengue (DENV). It is known that the main carbohydrate ligand recognized by the DC-SIGN receptor, which is responsible for mediating the interaction between these family of viruses with the surface of immune cells,^155–158^ is the high-mannose glycan (Man_9_GlcNAc_2_) with the mannosyl nonasaccharide Man_9_.^159^ To target the DC-SIGN lectin, the synthesis of mannosylated glycofullerenes and glycodendrofullerenes with 12 (41) or 36 (42–43) carbohydrate units conjugated to the fullerene scaffold using linkers of different length as antiviral agents against Ebola infection was described (Fig. 10).^153,154^ For this purpose, simple mannose or two mannose glycodendrons with different spacers between the central C_60_ core and the peripheral carbohydrate-substituted dendron were attached to the alkyne-substituted hexakis-adducts. The authors demonstrated the importance of multivalency in these systems with glycodendrofullerene 42, which features a longer linker and 36 mannose units, showing good activity (IC_50_ = 300 nM) when compared to glycofullerene 41 with 12 mannose residues (IC_50_ = 2000 nM). On the other hand, the most compact system 43, decorated with 36 carbohydrates but with a shorter linker, showed less activity (IC_50_ = 68 000 nM) than 41 (12 mannose motifs). The authors proposed that steric congestion, when shorter linkers were used, prevented accessibility of all glycans to interact with the DC-SIGN receptor and thus led to decreased binding affinities. These results highlighted that the length and flexibility of the linker unit and consequently the adequate accessibility of the ligands, plays a critical role in the interaction with surface receptors, and it is as important as the valency of the probe. In fact, the importance of the length of the linker in these type of macromolecules in biomedical applications was further confirmed by NMR spectroscopy studies.^160^ For compounds with same valency but different linkers, longer spacers make glycofullerene derivatives more dynamic and flexible, favouring the binding with the receptor. With this information in hand, glycofullerene oligomers 43–46 with 20, 30 and 40 mannose residues with long linkers were prepared as described above (Fig. 10).^161^ The ability of these oligomers to inhibit the infection of DC-SIGN Jurkat cells using pseudotyped Ebola viral particles as infectious agents were thus measured. It is important to highlight that all these glyconanostructures were not cytotoxic to cell lines at the concentrations used in the infection experiments, allowing their study of their potential to inhibit the viral infection. As expected, the compound 46 with a higher number of mannoses showed the best affinity for DC-SIGN in a mannose-dependent inhibition effect. Also, the tetravalent oligomer 46 with similar number of carbohydrates to the glycodendrofullerene 42 showed higher inhibitory effect, which can be accounted for by a topologically more favourable interaction.

**Fig. 11:**
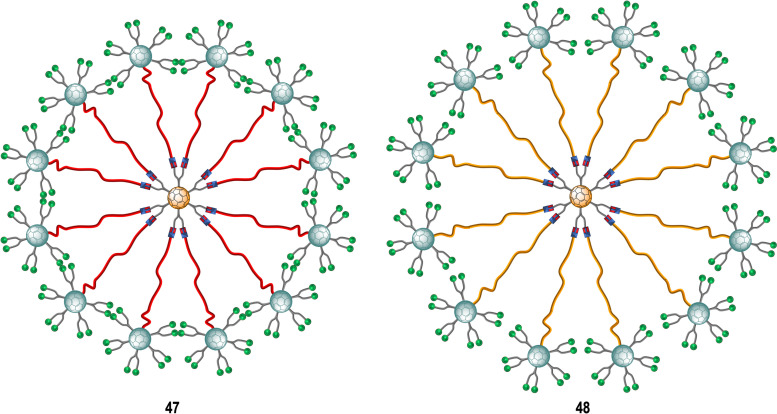
General representation of mannosylated superballs 47 (with a shorter linker, highlighted in red) and 48 (with a longer linker, highlighted in orange).

Subsequent studies focused on finding more effective inhibitors showed that dramatically increasing the valency and the size of the fullerene derivatives, led to an augmentation of the inhibitory activity against the Ebola virus. The same authors described the preparation of tridecafullerenes 47–48 (Fig. 11), so-called “superballs”.^162^ Using a combination of Bingel reaction and click chemistry, giant globular multivalent glycofullerenes were accessed.^152–154^ A C_60_ scaffold bearing 12 alkyne groups was decorated with 10 mannose-containing C_60_ units *via* CuAAC click chemistry in good yields, using CuBr·S(CH_3_)_2_ sodium ascorbate and metallic Cu in DMSO, to generate “sugar superballs” with a coating of 120 mannose subunits. The synthesis of these superballs was achieved in good yields *via* CuAAC click reaction employing. Moreover, a tridecafullerene coated with galactose was also prepared as negative control, since DC-SIGN is not able to recognize galactose. These complex molecules could be characterized by standard spectroscopic techniques such as NMR and FTIR spectroscopy, in addition to DLS, TEM and XPS.

To study the effect of glycan steric congestion on viral interactions, two different superballs featuring the same number of glycans but with either an ethylene glycol or an alkyl spacers between the peripheral carbohydrate-functionalized fullerenes and the fullerene central core were explored. These “giant” tridecafullerenes showed strong ability to inhibit the infection of DC-SIGN Jurkat cells using Ebola virus glycoprotein (EBOVGP) pseudotyped viral particles as infectious agents. It was found that both tridecafullerenes functionalized with mannose moieties exhibited very strong antiviral activity in the picomolar to nanomolar concentration range. Compound 48 (IC_50_ of ∼0.7 nM) was almost one order of magnitude more potent at inhibiting the infection process in comparison with the most compact tridecafullerene 7 (IC_50_ of ∼20 nM). As expected, the most flexible ethylene glycol-based linker (compound 48) allowed better accessibility and availability of the carbohydrates in the interaction with the corresponding receptor. These results were in agreement with those found for glycodendrofullerenes 42–43. Moreover, a multivalent effect was clearly observed when comparing glycodendrofullerenes coated with 12 (41) or 36 mannoses (42) with 48 (120 mannoses) with IC_50_ values for tridecafullerenes of up to three orders of magnitude higher (two if the mannoses number is taken in account). These results further confirmed how important the length and the flexibility of the linker unit between the core and the peripheral carbohydrates are as important as the number of glycans in mediating the viral infection. In fact, the importance of the length of the linker of new tridecafullerenes had already been highlighted in other applications such as in the photophysical behavior of these kind of macromolecules in water.^163^

Amphiphilic dendrofullerenes using fullerene C_60_ functionalized with two and four trivalent mannose glycodendrons were also explored to study the antiviral impact of different sizes and geometry of glycofullerenes.^164^ These glycodendrofullerenes with amphiphilic character in aqueous media self-assembled into spherical aggregates of micelle type as determined by scanning electron microscopy (SEM) and XRD and low angles X-ray scattering (SAXS) experiments. Additionally, these supramolecular aggregates showed a similar and uniform size for both glycodendrofullerenes, which could be associated by strong π–π interactions between fullerene C_60_ cores. Both micellar aggregates were capable of inhibiting DC-SIGN in the range of nanomolar concentrations in the same Ebola virus experimental model as previously mentioned.

**Fig. 12:**
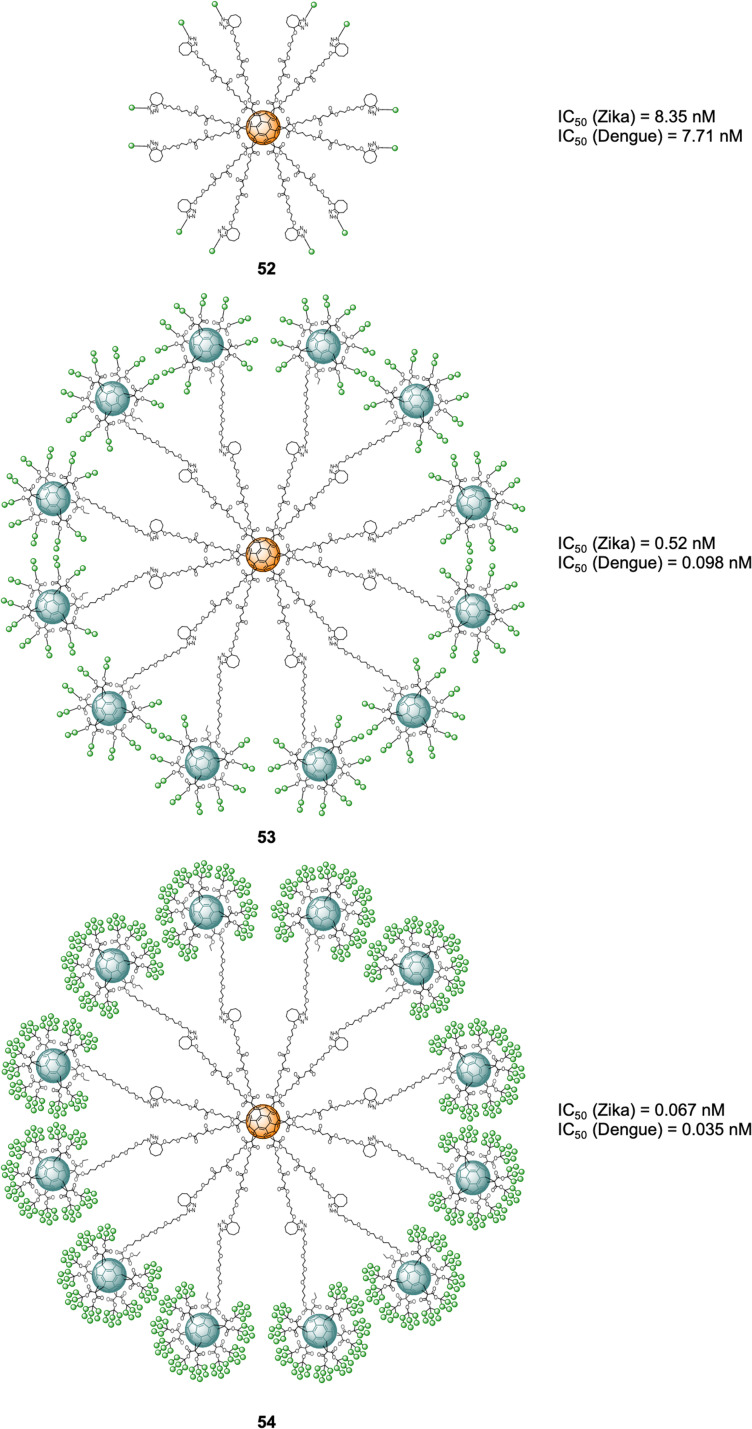
General structures of nanoballs 52–54 and IC_50_ values for nanoballs in inhibition studies Zika and Dengue-pseudotype virus.

More recently, examples of probes with an increased number of carbohydrates linked to a fullerene-based scaffold as well as branching points was described. Ground-breaking mannobiosylated tridecafullerenes, so-called ‘nanoballs’, with up to 360 α(1,2)mannobiosides functionalities were reported.^165^ It was found that replacing a single mannose as reported before with the disaccharides, increased the affinity to DC-SIGN by 3–4 fold. Initial approaches for the construction of glycofullerenes required high amounts of copper salts for the click reaction, which can be problematic in some instances. To address this, a Strain-Promoted Azide-Alkyne Cycloaddition (SPAAC) synthetic strategy based on post-functionalizable hexakis adducts of [60]fullerene was devised.^166,167^ Symmetric and asymmetric hexakis-adduct of C_60_ functionalized with twelve cyclooctyne groups were used to further carry out the SPAAC reaction with azide functionalised glycosides.^166^ In this manner, glycofullerenes functionalized with 12 galactoses (49, as the negative control, not shown), 12 mannoses (50, as the positive control, not shown) and either 12 (51) or 36 (52) α(1,2)mannobioside residues were assembled (Fig. 12). Moreover, nanoballs functionalised with 120 (53) and 360 (54) α(1,2)mannobiosides motifs were also prepared using this approach (Fig. 12). These examples represent quite a synthetic feat which is made possible by the application of SPAAC instead of the previously used CuAAC which requires cytotoxic copper, thus purification of the probes is easier and reactions proceed in higher yields and in lower reaction times, which are important considerations when using sophisticated building blocks such as mannobiosides or oligomannosides. The Martín's group evaluated the inhibitory efficiency of compounds 49–54 in a pseudotyped Zika (a positive single-strand RNA virus transmitted by mosquitos) and Dengue viral models employing Jurkat DC-SIGN cells (Fig. 12). The team found picomolar to nanomolar antiviral activity for nanoballs 52–54 (Fig. 12), with 54, which 360 carbohydrates on the surface the best of the series in terms of inhibiting both ZIKV and DENV (IC_50_ of 67 pM for ZIKV and IC_50_ of 35 pM for DENV). These results clearly show the multivalent effect with one order of magnitude enhancement on the IC_50_ inhibitory values as the number of carbohydrate moieties increase. Furthermore, it also validates the use of higher glycofullerene-based antivirals as effective probes to block carbohydrate receptors on the cell surface and inhibit the infection process. Moreover, this study opens the door to the development of new potential treatments against ZIKV by targeting carbohydrate–protein interactions since currently there are no effective therapies against this virus.

### 5.2. Glycofullerenes as probes in enzyme inhibition

Glycosidases are ubiquitous enzymes that cleave glycosidic bonds of glycoconjugates and oligosaccharides in nature. As a result, these important carbohydrate-modifying enzymes play an important role in the regulation of many protein/carbohydrate mediated processes^168,169^ and are attractive targets for the development of biomedical agents for the treatment of pathologiessuch as viral infections,^170^ cancer and tumour metastasis,^171,172^ influenza,^173^ diabetes,^174–176^ Alzheimer disease,^177^ lysosomal storage disorders^178^ and as antimicrobial agents.^179,180^ Unlike lectins which often feature multiple carbohydrate recognition domains, glycosyl-modifying enzymes such as glycosidases and glycosyltransferases are thought to generally have a single substrate-binding site which can bind to the carbohydrate substrate with high affinity and selectivity. Despite the common “monovalent substrate recognition” dogma, the development of multivalent platforms as glycosidase inhibitor has become a growing area of interest in glycoscience in the last few years, and inhibitors based on a wide range of core structures such as cyclodextrins,^181–184^ glucose,^185^ galactose,^185^ calix[4]arene,^185^ porphyrin,^185^ trehalose,^185^ micelles,^186^ cyclopeptoid cores,^187,188^ dextran polymers,^189^ and nanodiamonds,^190^ among others, have been described. For instance, iminosugar-fullerene conjugates with high valence have been shown to display strong binding enhancements to the target enzyme over the corresponding monovalent inhibitory motif (inhitope), demonstrating that multivalent platforms can potentially help enhance inhibitory activity.^186,191,192^

**Fig. 13:**
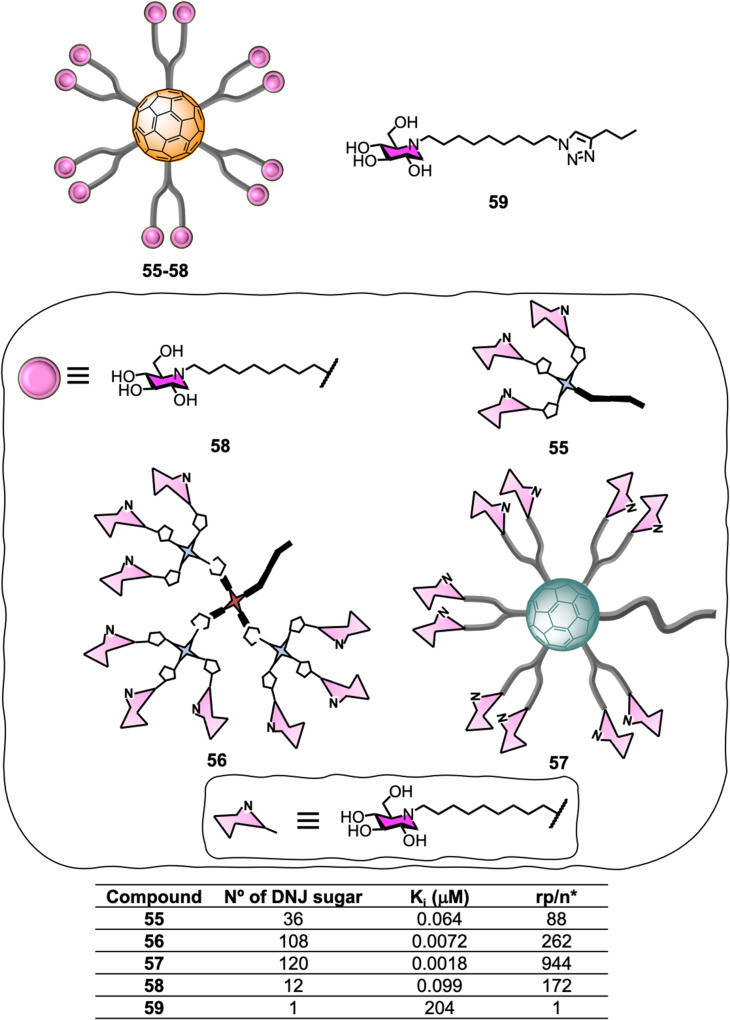
General representation of glycofullerenes 55–58 and monovalent iminosugar 59 and their corresponding α-Mannoside (JB α-man) inhibitory activities (*K*_i_). *rp = *K*_i_ (reference)/*K*_i_ DNJ-fullerene, *n* = number of inhitope units relative to the inhitope number in the reference.

Encouraged by the successful application of C_60_-scaffolds for antiviral applications, the synthesis of 1-deoxynojirimycin (DNJ), a well-known glycosidases inhibitor and inhitope for several enzymes of this class, functionalised C_60_-glycoconjugates, and their associated sp^2^-iminosugar glycomimetics have been reported as multivalent scaffolds as potential inhibitors against different glycosidases.^193–195^ Glycofullerenes decorated with DNJ iminosugars 55–56^193^ and the superball 57,^194^ were prepared *via* CuACC following as similar approach as described in Section 5.1 (Fig. 13). The nanoconstructs differ in the carbohydrate valency which is presented in a globular manner. As a structurally related monovalent control, iminosugarball 58^196^ decorated with 12 DNJ moieties and the corresponding monovalent DNJ iminosugar 59 were also included in the study for comparison (Fig. 13). It is important to highlight that carbohydrate-fullerene derivative 58 featuring 12 DNJ *via* six malonate linkers was one of the first examples of glycosidase inhibitors based on a multivalent scaffold.^196^

The inhibition profile of the C_60_-based DNJ glycoconjugates 55–57 was evaluated against a panel of commercial glycosidases including α-glucosidases (from yeast maltase and *A. niger* amyloglucosidades), almond β-glucosidades, α-galactosidades (from green coffee beans), β-galactosidase (from *E. coli*) and α-mannosidades (from Jack Bean α-Man) with some success, which was dependent on degree of multivalency.^193,194^ In particular, the teams of Mellet and Nierengarten centred their efforts on Jack Bean α-mannosidase (JB α-Man), known to be a glycosidase candidate for multivalent enzyme inhibitory (MEI) studies (Fig. 13). First, a three orders of magnitude enhancement of the glycosidase inhibitory effect was observed for first-generation glycodendrofullerene 55, which is loaded with 36 iminosugars, when compared to the corresponding monovalent compound. Moreover, 55 showed a similar *K*_i_ to the corresponding dodecavalent glycocluster 58. Second-generation glycodendrofullerene 56, with 108 DNJ motifs at the periphery, and 120-valent DNJ-coated superball 57, exhibited very potent inhibitory activity against JB α-Man with *K*_i_ values of 7.2 and 1.8 nM, respectively. A direct comparison with glycodendrofullerene 55, showed a one order of magnitude binding enhancement showing that increasing the number of DNJs on the scaffolds is beneficial for binding. Remarkably, giant iminosugarball 57 showed a 1 000 000-fold increase in inhibition potency when compared to monovalent compound 59 and a 55-fold enhancement against the corresponding dodecavalent mimic 58, which is well over a statistic effect.

In order to study the effect of MEI, the relative inhibition potency on a DNJ molar basis (rp/*n*) was analysed (Fig. 13). Whilst iminosugarballs 58 and 55 showed similar *K*_i_ values, the normalized relative inhibition potency for both probes was 172 and 88, respectively, which suggests that a higher inhitope density is slightly detrimental to relative potency. For derivative 56, which has a 108 valency, a three-fold rp/*n* value increase when compared to the corresponding first-generation analogue 55 was observed, clearly correlating to a multivalent effect. Interestingly, the MEI effect (*K*_i_) for 120-valent DNJ-superball 57 or 108-valent 56 resulted to be similar, suggesting that the inhitope density on multivalent scaffolds is also an important parameter that contributes to the inhibitory effect, thus combining these two aspects as a strategy, can lead to very potent inhibitors. In all cases, the inhibitors were competitive except for super-iminoball 57, which showed a mixed-type inhibition pattern, suggesting the scaffold can bind to both the enzyme and the enzyme-substrate complex, preventing substrate hydrolysis in both cases. These examples demonstrated for the first time multivalent probes can be used to inhibit α-mannosidase activity even when the active site is fully occupied. These results are also in line with previous reports of homogeneously or heterogeneously decorated fullerene probes with either carbohydrates or sp^2^-iminosugar glycomimetics^195,197–200^ for β-galactosidase inhibition.^188,195,201^

**Fig. 14:**
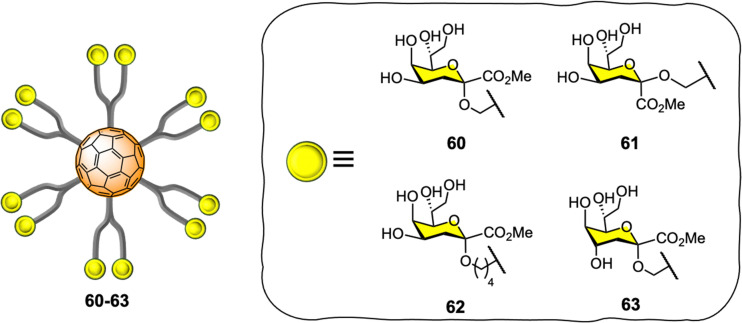
General representation of compounds 60–63.

Vicent and co-workers expanded the application of glyco-fullerene probes for multivalent enzyme inhibition to other enzymatic systems such as glycosyltransferases.^202–204^ The authors showed the efficacy of the mannopyranose core structure of bacterial l,d-heptoside glycofullerenes as glycosyltransferase inhibitors in the low micromolar range (IC_50_ = 7–45 μM).^203^ In 2016, the same team disclosed the synthesis of keto-deoxyoctulosonate (Kdo) glycofullerenes 60–63 (Fig. 14) and the enzymatic assays against therapeutically relevant bacterial heptosyltransferase I (WaaC),^204^ which is an important bacterial glycosyltransferase involved in the biosynthesis of bacterial lipopolysaccharides (a key structural component of the bacterial membrane) and display two recognition sites.

The novel Kdo glycofullerenes 60–63 displayed very potent inhibition activity, much more active as inhibitors and affine for glycosyltransferase than the previously described l,d-heptosides glycofullerene derivatives, with compound 60 being the best inhibitor with a *K*_i_ = 0.14 μM, which is particularly remarkable and with a potency rarely observed with glycosyltransferases using monovalent inhibitors. Moreover, the inhibitory efficiency of the probes toward the WaaC enzyme was linked to the absolute configuration of the C-1 and C-4 substitutes in the Kdo moiety, while the linker length between the carbohydrate and platform did not seem to have an effect. It was also found that that no significant multivalent effect when compared to monovalent analogues was observed for glycofullerenes 60–63, as calculated by the affinity enhancement of a ligand when presented in a multimeric fashion, based on normalized IC_50_ values. Mechanistically, it was postulated that glycofullerenes interacted only with the glycosyl acceptor binding site, which is more exposed and easily accessible than the donor site. These new discoveries open the door to a new class of inhibitors for glycosyl-modifying enzymes where scaffolds such [60]fullerene or others carbon nanoforms can be used to tune the selectivity and potency of monovalent inhibitors. Moreover, the concept of multivalent enzyme inhibition could be applied to other kinds of enzymes which are membrane bound on the surface of cells or viruses.

### 5.3. Glycofullerenes as probes in anti-bacterial applications

**Fig. 15:**
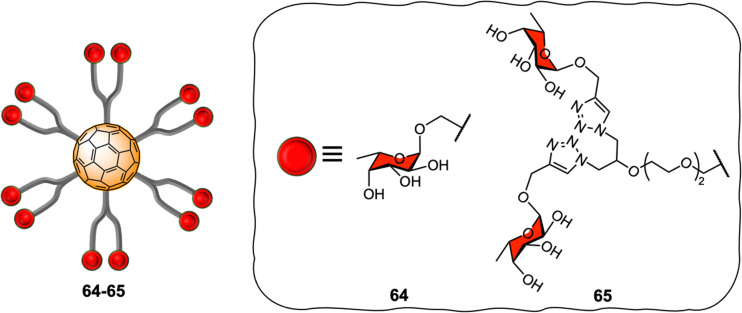
General representation of compounds 64–65.

[60]fullerene molecules have also been used as multivalent platforms for applications as anti-bacterials. The first examples of such applications targeted the interaction between FimH and PA-IL (a bacterial lectin from *P. aeruginosa*) and their corresponding glycan cell surface ligands, using water-soluble mannosylated and galactosylated dodecavalent-adducts of fullerene, respectively.^205,206^ Latter, the Buffet group reported the preparation of a series of fucofullerenes 64–65 containing up to 24 fucose residues which could be used against two bacterial lectins namely LecB from *P. aeruginosa* and RSL from *R. solanacearum* (Fig. 15).^207^

C_60_(Fuc)_24_65 appeared to be the best inhibitor with a *K*_D_ in nanomolar range, measured by isothermal titration calorimetry (ITC) experiments for both LecB and RSL reported to date, showing a binding affinity enhancement when multimeric ligands were used and further demonstrating the potential of these types of scaffolds as anti-adhesive therapeutics.^9,208,209^ Interestingly, when taking into account the efficiency per ligand epitope, and comparing with a monovalent ligand, RSL showed that the multivalent effect was more significant for RSL than for LecB. The authors attributed the different lectin behaviour to differences in topology of both lectins, since RSL features six binding pockets on the same face of the protein, while LecB's four binding pockets were far apart and did not allow for facile multivalent interactions using these probes. Indeed, in the latter case, only the simultaneous binding of two carbohydrate units from the same fucofullerene to two different LecB protein was possible. The authors concluded that any observed multivalent effects can be attributed exclusively to aggregation effects, as confirmed by ITC experiments. On the other hand, for the RSL lectin, the authors proposed that the binding enhancement is likely due to a chelate binding effect between fucofullerenes and the lectin, although due to steric hindrance around the fucofullerene probes, only a small number of glycan epitopes are able to bind at any given time.

### 5.4. Glycofullerenes as probes in anticancer applications

Monofunctionalized glycofullerene derivatives featuring one or two carbohydrate units have been extensively exploited as PDT photosensitizers against cancer cells owing to their ability to either act as ROS generators, or radical scavengers, depending on the presence or absence of light.^149^ However, a very few examples of hexa-substituted glycofullerenes as anticancer agents have been reported in the literature to date. Very recently, Gallego *et al.*^210^ reported the synthesis a fully hexa-substituted fullerene coated with a peptide mimetic of the natural ligand sLe^X^ for the specific recognition of the E-selectin surface receptors in the membrane of endothelial cell with potential application in cancer diseases.

**Fig. 16:**
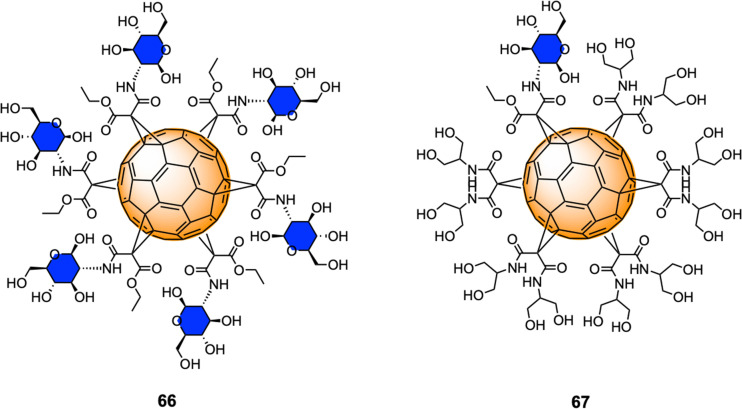
Structures of glycofullerenes 66–67.

Serda *et al.*^211^ have employed glycofullerenes as non-receptor inhibitors of tyrosine kinases, which are cytosolic enzymes able to regulate cell growth, proliferation, differentiation, adhesion, migration and apoptosis and are involved in pancreatic cancer. Glycofullerenes 66–67, decorated with d-glucosamine motifs conjugated *via* the C-2 amine to the central scaffold and thus exhibiting a free anomeric OH (Fig. 16) were tested as potential inhibitors of non-receptor tyrosine kinases including the BRK, ABL1, BTK and Src family kinases (CSK, Fyn A, Lck, Lyn B and Src). Glycocluster 66, with 6 glycan units, showed a higher inhibitory activity than mono-decorated 67 for Src family kinases, as well as for ABL1, BRK, and BTK kinases. Moreover, glycofullerene 66 exhibited the lowest IC_50_ values for Fyn A and BTK proteins and could selectively modulate the activity of both enzymes, which are promising targets for anti-cancer therapies. SDS-PAGE electrophoresis studies suggested that the observed selectivity could be attributed to the formation of a protein corona around 66. Glycofullerenes 66 and 67 induced autophagy and disrupted redox balance, triggering the upregulation of repair systems and disrupted Fyn and BTK protein levels in both pancreatic cancer cell lines, whilst being non-toxic for pancreatic cancer cells (PANC-1 and AsPC-1).

Previous work from the same authors demonstrated the cellular uptake of C_60_(GlcNAc)_6_66 in pancreatic stellate cells (PSCs) using confocal microscopy,^212^ which are involved with the establishment of dense stroma regions in cases of advanced pancreatic adenocarcinoma, and it was found that compound 66 was predominantly accumulated in the nucleus of PSCs and displayed high selectivity and strong photodynamic cytotoxic behavior, when irradiated with blue and green light.

More recently, Serda and co-workers also reported the cellular proliferation, glycolysis rate, migration and metabolic activity of PANC-1 cells after incubations with different concentrations of glycofullerene 66.^213^ Interestingly, at low concentrations of this glycofullerene, a decrease in the rate of PANC-1 cell proliferation and an increase in the metabolic activity were found, suggesting changes in the glucose metabolism during cell differentiation induce upon inhibition. Moreover, glycofullerene 66 did not show any influence on various other cancer cellular events and glycolysis. There results suggested that glycofullerene 66 could be considered as a promising drug delivery vehicle for treating pancreatic cancer.

These results represent the first example of [60]fullerene glyconanomaterials as selective non-receptor tyrosine kinases inhibitors, and further demonstrated the potential of these type of nanomaterials as dual cancer nanotherapeutics and photodynamic therapy agents and drug delivery vehicles for pancreatic cancer.

## 6. Concluding remarks

During the last few decades, many advances of glycoscience have help elucidate the fundamental roles that carbohydrates play in nature. The search for tools to study disease-relevant carbohydrate-mediated processes, such as viral infection, cancer or antibacterial inhibition, is of great importance not just in the area of glycobiology but also in the biomedical and medicinal chemistry fields. Within this context, the scientific community has focused its efforts on the development of multivalent glycoprobes to allow the study of carbohydrate–protein interactions relevant to physiological and pathological processes. In particular, biocompatible carbon nanoforms appear to be ideal candidates for the generation of 2D and 3D scaffolds that can present multiple copies of a glycan (natural or synthetic ones) with tuneable characteristics. Graphene and their corresponding derivatives, carbon nanotubes, carbon dots, and fullerenes, are very promising and biocompatible scaffolds for the multivalent presentation of carbohydrates. It has now been acknowledge that not only multivalency, but also the nature, spatial disposition (including flexibility and chemical nature of linkers) and distribution of carbohydrates around a carbon nanosized scaffold, as well as the size and shape of carbon nanoplatform, are key parameters to be considered for the successful interaction of glycans with their corresponding cellular receptors. It has become clear that the careful choice of the carbon nanosized structures as scaffold is key when designing multivalent glycoconjugates. The rapid growing field of carbon-based nanomaterials has led to the development of a great range of novel carbon nanoforms, such as carbon nanohorns, carbon nanoonions, peapods, carbon nanotori, carbon nanobuds and more recently 2D graphene quantum dots (GQDs) and carbon quantum dots (CQDs), which could also be explored as potential scaffolds for the multivalent presentation of carbohydrates. For example, it is worth highlighting that the highly symmetric glycofullerene allows the controlled chemical modification of the carbon nanocore, which permits the synthesis of homogeneous and well-defined multivalent glycoconjugates with high purity that is reflected in perfectly oriented glycosidic copies in the spatial environment (in a globular manner) allowing a direct comparison between different glycans. This is in contrast to polydisperse 3D carbon dot nanomaterials, where carbohydrate surface coating and density is less well defined. On the other hand, glyco-SWCNTs and -MWCNTs can mimic the shape or surface of filamentous structures of specific viruses, such as Ebola virus, but still represent non-homogenous nanoparticles. Taking into account the enormous variety of potential carbohydrate ligands, both in terms of their structure (*e.g.*, monosaccharides, disaccharides, polysaccharides, *etc.* and composition) and surface presentation (*e.g.*, defined dendrimers, degree of surface functionalization), a wide range of combinations of multivalent nanosized glycoconjugates are required to better understand the nature of protein/carbohydrate interactions at a molecular level. In this context, methods to efficiently select the correct ligand(s) and adequate surface presentation on different scaffolds have the potential to explore all the chemical space available by furnishing a variety of lead hybrid molecules with high specificity and efficiency for bespoke applications. However, despite the many efforts, controlled carbohydrate functionalization and distribution on a given nanostructures and their structural characterization still present a significant scientific challenge. Considering the recent innovative developments in carbon nanoforms, synthetic carbohydrate chemistry, glycobiology and nanotechnology, further innovations with regards to the carbohydrate ligand and carbon-nanosized structures will help the progress of this field. There is still a lot of room for new advances in this active area of research, and great progress is expected in the near future with the synergistic combination of all these important fields.

## Conflicts of interest

There are no conflict of interest to report, however MCG wishes to declare that she is co-founder and co-director of CDOTBIO Ltd.

## Supplementary Material
